# The treatment of sarcoptic mange in wildlife: a systematic review

**DOI:** 10.1186/s13071-019-3340-z

**Published:** 2019-03-13

**Authors:** Madeleine L. Rowe, Pam L. Whiteley, Scott Carver

**Affiliations:** 1 Faculty of Veterinary and Agricultural Science, The University of Melbourne, Werribee Campus, Werribee, VIC 3030 Australia; 2 School of Biological Sciences, University of Tasmania, Sandy Bay, Hobart, Australia

**Keywords:** Wildlife, Sarcoptic mange, *Sarcoptes scabiei*, Treatment, Control

## Abstract

**Background:**

Sarcoptic mange, caused by the *Sarcoptes scabiei* mite, is an infectious disease of wildlife, domestic animals and humans with international importance. Whilst a variety of treatment and control methods have been investigated in wildlife, the literature is fragmented and lacking consensus. The primary objectives of this review were to synthesise the diverse literature published on the treatment of sarcoptic mange in wildlife from around the world, and to identify the qualities of successful treatment strategies in both captive and free-roaming wildlife.

**Methods:**

A systematic search of the electronic databases CAB Direct, PubMed, Scopus, Web of Science, EMBASE and Discovery was undertaken. Data pertaining to study design, country, year, species, study size, mange severity, treatment protocol and outcomes were extracted from eligible studies and placed in a table. Following data extraction, a decision tree was used to identify studies suitable for further analysis based on the effectiveness of their treatment protocol, whether they were conducted on captive or non-captive wildlife, and the quality of their post-treatment monitoring period.

**Results:**

Twenty-eight studies met our initial inclusion criteria for data collection. Of these studies, 15 were selected for further analysis following application of the decision tree. This comprised of 9 studies on captive wildlife, 5 studies on free-living wildlife and 1 study involving both captive and free-living wildlife. Ivermectin delivered multiple times *via* subcutaneous injection at a dose between 200–400 µg/kg was found to be the most common and successfully used treatment, although long-term data on post-release survival and re-infection rates was elusive.

**Conclusions:**

To our knowledge, this review is the first to demonstrate that multiple therapeutic protocols exist for the treatment of sarcoptic mange in wildlife. However, several contemporary treatment options are yet to be formally reported in wildlife, such as the use of isoxazoline chemicals as a one-off treatment. There is also a strong indication for more randomised controlled trials, as well as improved methods of post-treatment monitoring. Advancing this field of knowledge is expected to aid veterinarians, wildlife workers and policy makers with the design and implementation of effective treatment and management strategies for the conservation of wildlife affected by sarcoptic mange.

**Electronic supplementary material:**

The online version of this article (10.1186/s13071-019-3340-z) contains supplementary material, which is available to authorized users.

## Background

Sarcoptic mange, caused by the mite *Sarcoptes scabiei*, is a globally-distributed, infectious disease of wildlife that is emerging in some species [1] and has been reported in greater than 100 species of mammals [2]. The sarcoptic mite burrows deep into the epidermis, causing inflammation, intense pruritis and, in advanced cases, a perturbed skin barrier that may result in death secondary to infection, dehydration and impaired thermoregulation [3, 4]. In highly susceptible populations, the mite has the capacity to spread rapidly, reduce reproduction and cause mass mortality events [5–7]. The death of only a few reproducing adults may have significant consequences for threatened or isolated populations [8], especially when combined with other threatening processes, such as habitat destruction [9, 10]. Consequently, the treatment and control of sarcoptic mange may play an important role in conservation. Whilst comprehensive reviews have been published on the pathogenesis and epidemiology of sarcoptic mange in wildlife [2, 11], to our knowledge no review has focussed specifically on methods of treatment and their long-term outcomes.

Thus, this review aims to systematically review primary articles on the treatment of sarcoptic mange in wildlife, with a focus on the qualities of successful treatment strategies and their long-term outcomes. The review also aims to highlight research deficiencies and to discuss when treatments may or may not be warranted. Greater synthesis and consensus in this field of knowledge is expected to assist veterinarians, wildlife workers and policy makers with the design and implementation of effective treatment and management strategies for the conservation of wildlife affected by sarcoptic mange.

## Methods

This systematic review was conducted in accordance with the definition provided by the PRISMA (Preferred Reporting Items for Systematic Reviews and Meta-Analyses) statement: ‘… a review of a clearly formulated question that uses systematic and explicit methods to identify, select and critically appraise relevant research, and to collect and analyse data from the studies that are included in the review’ [12] (see PRISMA checklist in Additional file 1: Table S1). We did not register the protocol for this review.

### Search strategy

A systematic search of six electronic databases was conducted between May and August 2017. CAB Direct (1973–2017), PubMed (1951–2017), Scopus (1995–2017), Web of Science (1900–2017), EMBASE (1946–2017) and Discovery (1401–2017) were searched with no date restrictions but with results limited to those published in English, as permitted by the databases. The search strategy included the following key terms (and possible variants of the terms including alternate spellings): Sarcoptic mange, scabies AND wildlife, population, native, indigenous, local, animal, free-roaming, free-ranging, undomesticated AND treatment, therapy, cure, medicate, rehabilitate, remedy. Terms were searched in title, keyword and abstract (as permitted by the databases). The full search strategy is included in Additional file 2: Text S1.

### Inclusion and exclusion criteria

**Fig. 1:**
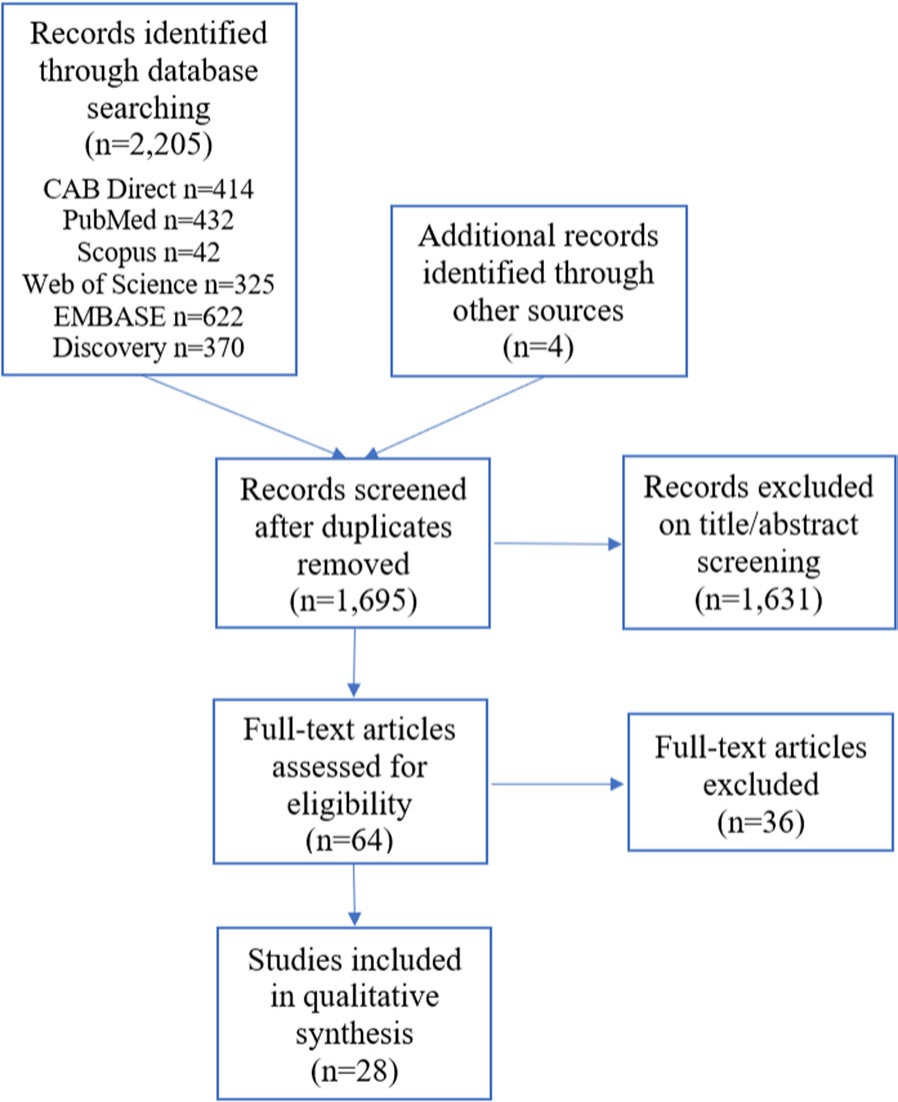
Flow diagram illustrating the selection of studies to be included in the review

Studies were exported into EndNote X6 and duplicates were removed. The inclusion/exclusion selection process is illustrated in Fig. 1. Titles and abstracts were screened for relevance, and irrelevant research was excluded based on the following exclusion criteria: (i) any paper not in the English language or published in full-text; (ii) any reviews (although reviews specific to sarcoptic mange in wildlife were retained for backwards and forwards searching); or (iii) any paper that did not refer to the treatment of sarcoptic mange in wildlife in its title or abstract. Studies already known to the authors were also considered for inclusion, based upon the criteria reported above. Reference and citation lists of relevant studies and reviews were screened to identify additional articles, which were subject to the same criteria as results from the database searches. This process continued until no further research was obtained. Finally, the entire manuscript was evaluated. Papers were eligible for inclusion if they described the therapeutic treatment of a wildlife species diagnosed with sarcoptic mange. Treatment refers to ‘… medical care given to a patient for an illness or injury’, as defined by the Oxford English Dictionary [13]. Wildlife refers to ‘… feral animals, captive wild animals and wild animals.’, where a wild animal is ‘… an animal that has a phenotype unaffected by human selection and lives independent of direct human supervision or control’ and a captive wild animal refers to an animal that has ‘… a phenotype not significantly affected by human selection but that is captive or otherwise lives under direct human supervision or control, including zoo animals and pets’, as defined by the OIE [14]. For the purpose of this study, articles on feral animals or wildlife not infected with *S. scabiei* were excluded from analysis. Articles that involved the treatment of *S. scabiei* in both wild and domestic animals were retained. Where there was any uncertainty regarding the inclusion of a study, the opinion of a second reviewer was sought.

### Data extraction

Data were carefully extracted from eligible studies by a single independent reviewer under the following headings: study design, year and country of publication; taxonomic family and species of wildlife studied; number of animals treated; treatment environment (i.e. in the wild *versus* in captivity); severity of infection prior to treatment; treatment protocol (i.e. drug, dose, delivery method, number of doses delivered, and treatment interval between doses); and outcomes (see the complete data extraction table in Additional file 3: Table S2). Where not explicitly stated within studies, the degree of mange severity prior to treatment (i.e. mild, moderate or severe) was extrapolated from descriptions of the severity and distribution of mange lesions over the body of infected animals. Where not explicitly stated, study design was extrapolated using definitions from an authoritative textbook [15]. Outcomes were expressed as the percentage of animals that recovered with treatment (i.e. the treatment recovery rate), and adverse side effects were also documented.

### Quality assessment

**Fig. 2:**
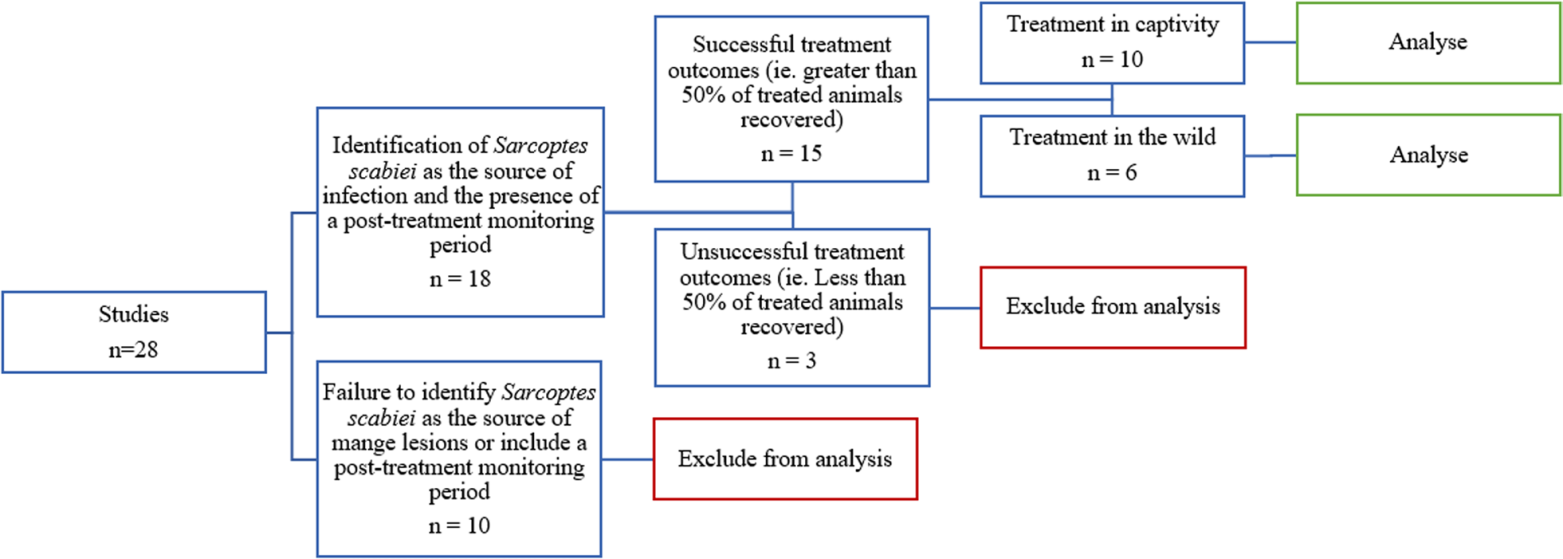
Decision tree illustrating the selection of studies for further analysis

Following the initial data collection, a decision tree was used to determine whether eligible studies were suitable for further analysis (Fig. 2). Studies that failed to identify *S. scabies* as the source of infection prior to treatment, or which did not document treatment outcomes or a post-treatment monitoring period were excluded from further analysis. The remaining studies were classified into two arbitrary categories: successful treatments (where greater than 50% of the animals treated for sarcoptic mange recovered following treatment) and unsuccessful treatments (where less than 50% of treated animals recovered following treatment). Successful studies were divided according to whether they involved the treatment of captive or free-roaming wildlife (otherwise referred to as non-captive, or free-ranging wildlife). They were then assessed on the severity of the animals’ mange prior to treatment, the treatment protocol, and the duration and outcome of post-treatment monitoring. A monitoring period was defined as a specified length of time for observing the process of recovery of one or more animals after delivery of the final medication in a treatment protocol.

As there is a dearth of literature in this area, all papers relevant to the subject were included in this systematic review, many of which were case reports and other forms of non-prospective or randomised studies. Therefore, we agree with the concern that much of the data could be subject to bias and have acknowledged this in the discussion. No formal statistical or meta-analysis was carried out and analysis to investigate statistical heterogeneity or publication bias was not performed because most of the studies were descriptive case series.

## Results

### General results and study design

A total of 2205 publications were retrieved from the database search. Duplicates were removed through Endnote X6, leaving 1687 results. Following the screening process, a total of 28 unique and relevant studies were reported in this systematic review (see Additional file 3: Table S2). Seventeen studies were case reports or series, seven were non-randomised controlled trials, two were cohort studies, and two were cross-sectional studies.

#### Year and location

This review looks at research on the treatment of sarcoptic mange in wildlife dating from the 1970s. Most studies were published following the year 2000, although in several cases the research start date was many years earlier than this. The majority of studies were undertaken in Australia, Europe and Africa (Table 1).

**Table 1:** The number of studies selected for data collection from each continent/country

Continent	No. of studies/continent	Country	No. of studies/country
Australia	7	Australia	7
Europe	7	Spain	4
		England	1
		Italy	1
		Croatia	1
Africa	5	Kenya	2
		Uganda	2
		Zambia	1
Asia	4	India	1
		Israel	1
		Japan	1
		Korea	1
North America	4	USA	4
South America	1	Peru	1

#### Animal families and species

**Fig. 3:**
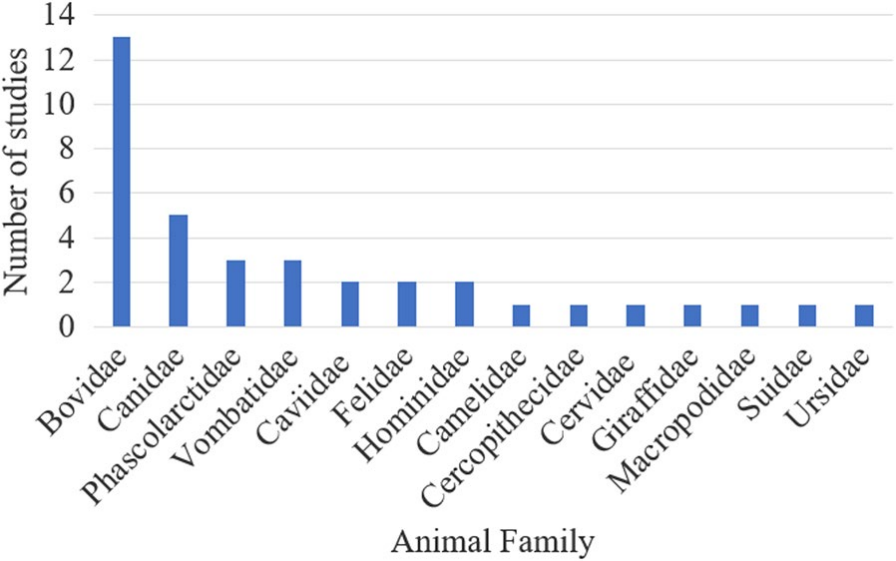
Histogram illustrating the taxonomic families of wildlife included in the review

Across the 28 primary articles, 30 species of wildlife, comprising 14 different taxonomic families of mammals, were treated for sarcoptic mange. Two studies treated greater than one species of wildlife [16, 17] and one of these studies, by Gakuya et al. [17], involved the treatment of domestic and wild animals as part of the management of sarcoptic mange in a complex wildlife/livestock system. The most commonly studied families were the Bovidae and Canidae (Fig. 3). As some studies involved the treatment of more than one animal family, the cumulative total for ‘Number of studies’ in Fig. 3 is greater than 28.

#### Adverse side effects of treatments

Four studies documented adverse side effects following administration of subcutaneous ivermectin or adjunctive therapies. Side effects included severely loose stools [18], profuse watery diarrhoea [9] and death [19, 20]. Of the remaining 24 studies, only three studies specifically monitored for or stated an absence of deleterious side effects following treatments [21–23]. Adverse side effects were either absent or failed to be documented in the other studies.

### Additional analyses

Quality assessment using a decision tree left 15 studies suitable for further analysis. This comprised of nine successful studies involving captive wildlife with a post-treatment monitoring period, five successful studies involving free-living wildlife with a post-treatment monitoring period, and one successful study describing the treatment of both captive and non-captive wildlife [24]. This study was subsequently included in both of these analysis groups; hence the cumulative number of studies under the captive and free-ranging treatment group headings in Fig. 2 equates to 16 and not 15.

#### Successful studies on free-ranging wildlife with a post-treatment monitoring period

Fluralaner, ivermectin, amitraz and phoxim were used as therapeutic acaricides in the ten studies involving the treatment of sarcoptic mange in captive wildlife (Table 2). The most commonly used therapeutic agent was ivermectin, featuring in nine out of the ten studies. Ivermectin was delivered by manual subcutaneous injection or remote rifle darting and at a dose ranging between 200–400 µg/kg.

**Table 2:** Summary of successful studies on captive wildlife with a post-treatment monitoring period

Treated species and reference	No. of animals treated	Severity^a^	Treatment^b^				Duration of post-treatment monitoring
			Drug(s) administered	Dose & delivery method	No. of doses	Treatment interval between doses	
American black bear (*Ursus americanus*), Van Wick et al. (2018) [21]	1	Severe	a) Fluralaner; b) Lactated Ringer’s solution; c) Hydrogenated iron	a) 44 mg/kg PO; b) 40 ml/kg SC; c) 10 mg/kg IM	a) 1; b) 1; c) 1	na	13 weeks
Mara (*Dolichotis caviae*), Kim et al. (2015) [25]	2/16 (i.e. clinically affected individuals)	Moderate	a) Amitraz; b) Prednisolone	a) 0.025% wash; b) 2 ml IM	a) 4; b) 4	a) 7 days; b) 7 days	
	16/16 (i.e. colony-wide treatment)	Mild to moderate	a) Ivermectin; b) Prednisolone	a) 400 µg/kg SC; b) 2 ml IM	a) 4; b) 4	a) 7 days; b) 7 days	104 weeks
Raccoon dog (*Nyctereutes procyonoides*), Kido et al. (2014) [27]	68	Moderate to severe	Ivermectin	400 µg/kg SC	2	14 days	18 weeks on average
	157	Moderate to severe	a) Ivermectin; b) Cephalexin; c) Lactated Ringer’s solution	a) 400 µg/kg SC; b) 20 mg/kg PO; c) Intravenous	a) 3; b) 14; c) 1	a) 14 days; b) 12 hours; c) na	18 weeks on average
Southern hairy-nosed wombat (*Lasiorhinus latrifrons*), Ruykys et al. (2013) [24]	2/5 (i.e. captive wombats)	Severe	Ivermectin	200 µg/kg SC	1	na	7–10 weeks, depending on the animal
African buffalo (*Syncerus caffer*), Munang’Andu et al. (2010) [26]	77	Mild to severe	Ivermectin	200 µg/kg SC	4	30 days	6 weeks
Roe deer (*Capreolus capreolus*), Menzano et al. (2008) [20]	3	Moderate to severe	Ivermectin	300 µg/kg SC	3	15 days	52 weeks
Bare-nosed wombat (*Vombatus ursinus*), Skerratt et al. (2003) [28]	2/7 (i.e. Experiment one)	Mild to severe	a) Ivermectin; b) Procaine penicillin; c) Benzaine penicillin	a) 300 µg/kg SC; b) 15 mg/kg IM; c) 11 mg/kg IM	a) 3; b) 1; c) 1	a) 10 days; b) na; c) na	22 weeks
	7/7 (i.e. Experiment two)	Mild to severe	a) Ivermectin; b) Procaine penicillin; c) Benzaine penicillin	a) 300 µg/kg SC; b) 15 mg/kg IM; c) 11 mg/kg IM	a) 3; b) 1; c) 1	a) 10 days; b) na; c) na	8 weeks
	3/7 (i.e. wombats whose mange recurred in Experiment two)	Mild	Ivermectin	300 µg/kg SC	3	10 days	18 weeks
Iberian ibex (*Capra pyrenaica*), Leon-Vizcaino et al. (2001) [23]	3	Moderate	Ivermectin	400 µg/kg SC	1	na	8 weeks
	3	Moderate	Ivermectin	400 µg/kg SC or superficial IM by rifle dart	1	na	8 weeks
	3	Moderate	Ivermectin	200 µg/kg SC	1	na	8 weeks
	3	Severe	Ivermectin	200 µg/kg SC	2	14 days	6 weeks
	3	Severe	Ivermectin	400 µg/kg SC	2	14 days	6 weeks
	17	None to mild	a) Phoxim; b) Ivermectin	a) 500 mg/l topical spray; b) 400 µg/kg SC	a) 1; b) 1	na	45 weeks
	32	Mild to moderate	a) Phoxim; b) Ivermectin	a) 500 mg/l topical spray; b) 200–400 µg/kg SC or superficial IM by rifle dart	a) 1; b) 3	a) na; b) 14 days	45 weeks
Chamois (*Rupricapra pyrenaica*), Lavin et al. (2000) [51]	2	Mild to moderate	Ivermectin	200 µg/kg SC	2	15 days	2 weeks
Red fox (*Vulpes vulpes*), Little et al. (1998) [52]	5	Severe	Ivermectin	400 µg/kg SC	2	14 days	8–15 weeks, depending on the animal

^a^Severity of mange prior to treatment

^b^Alphabetized bullet points indicate different medications delivered concurrently

*Abbreviation*: na, not applicable

Ivermectin was delivered between 1–4 times, with an average of 2.3 times and a median of 2 times. The interval of time between successive ivermectin treatments ranged from 7–15 days and was an average of 13.9 days and a median of 14 days. There was no consensus between the severity of infection and the therapeutic dose of ivermectin delivered. For instance, in some studies, mildly-diseased individuals were treated with 400 µg/kg subcutaneous ivermectin [23, 25], whereas in other studies, severely-diseased animals were treated with only 200 µg/kg [24, 26]. Dose did not appear to affect the overall success of treatment (i.e. the treatment recovery rate), although it may have influenced the speed of recovery. For instance, in a study by Leon-Vizcaino et al. [23], moderately-diseased Iberian ibex (*Capra pyrenaica*) that received a single dose of 400 µg/kg subcutaneous ivermectin experienced a reduction in the number of live mites on skin scrapings faster than moderately-diseased Iberian ibex that received a single dose of 200 µg/kg. Another finding was that the recovery rate of large numbers of infected animals treated simultaneously increased with subsequent treatments. For instance, in a study by Munang’Andu et al. [26], mange was eliminated from 54.5% of a population of moderately-diseased African buffalo calves (*Syncerus caffer*) after a single treatment with ivermectin *versus* 100% of moderately-diseased calves after two treatments. Furthermore, severely-diseased buffalo calves required three treatments for complete elimination of infection. Another factor that was positively associated with the success of treatment was concurrent administration of supportive therapy. For example, in the study by Kido et al. captive raccoon dogs (*Nyctereutes procyonoides*) that received ivermectin, antibiotics and intravenous fluids had a significantly higher rate of recovery than raccoon dogs that received ivermectin alone (61.1 *versus* 42.6%, respectively) [27].

Post-treatment monitoring periods ranged from 2–104 weeks and were an average of 20.8 weeks and a median of 8 weeks. Of the animals intended for release into the wild following treatment, only one study documented post-release survival outcomes and no studies documented reinfection rates. Rather, the studies monitored disease status up until the point of release from captivity and then stopped, or the animals remained in captivity for the duration of their lifespan. Two studies documented a recrudescence of the clinical signs of mange during their post-treatment observational period in captivity and required additional treatments to eliminate infection [25, 28]. The only study that attempted monitoring past the point of release from captivity involved the treatment of an American black bear with oral fluralaner [21]. The bear was monitored in captivity for 13 weeks, then released and tracked *via* GPS. The bear has since ‘remained active’, although its mange status has not been visually evaluated.

#### Successful studies on free-ranging wildlife with a post-treatment monitoring period

Ivermectin, amitraz, deltamethrin and ‘tebrub’ were used as therapeutic acaricides to treat sarcoptic mange in the studies on free-ranging wildlife (Table 3). Ivermectin featured in all studies. It was delivered *via* manual subcutaneous injection, remote rifle darting or orally in food and at a dose ranging between 170–800 µg/kg. Ivermectin was delivered between 1–7 times, with an average of 1.8 times and a median of 1 time. This is less than the average and median number of treatments delivered to the animals in the studies involving captive wildlife (2.3 and 2 times, respectively). Again, there was no consensus between the dose of ivermectin administered and the severity of infection of the animals treated. For example, in one study, a mildly-diseased mountain gorilla received a dose of 670 µg/kg subcutaneous ivermectin to treat mange, whereas a moderately to severely-diseased mountain gorilla received a considerably smaller dose of 170 µg/kg; both recovered [9].

**Table 3:** Summary of successful studies on non-captive wildlife with a post-treatment monitoring period

Treated species and reference	No. of animals treated	Severity^a^	Treatment^b^				Duration of post-treatment monitoring
			Drug(s) administered	Dose & delivery method	No. of doses	Treatment interval between doses	
Southern hairy-nosed wombat (*Lasiorhinus latrifrons*), Ruykys et al. (2013) [24]	3/5 (i.e. free-roaming wombats in the study)	Mild or severe	Ivermectin	200 µg/kg SC	1	na	10–14 weeks, depending on the animal
Cheetah (*Actinonyx jubatus*) and others, Gakuya et al. (2012) [17]	Unknown	Mild to severe	Ivermectin	200 µg/kg SC	1	na	30–74 weeks, depending on the animal
Wild boar (*Sus scrofa*), Rajkovic-Janje (2004) [31]	750	Unreported	Ivermectin	100 µg/kg PO	7	1 day	4 weeks
Bare-nosed wombat (*Vombatus ursinus*), Skerratt et al. (2004) [29]	5	Mild to moderate	a) Ivermectin; b) Amitraz	a) 400–800 µg/kg SC; b) 0.025% topical wash	a) 2; b) 1	a) 28 days; b) na	1 week
	1/5 (i.e. one retreated)		Ivermectin	800 µg/kg SC injection	2	10 days	11 weeks
Mountain gorilla (*Gorilla beringei beringei*), Kalema-Zikusoka et al. (2002) [9]	3	Moderate to severe	a) Ivermectin; b) Long-acting streptopenicillin; c) Oxytetracycline spray; d) Ferrum 10% + vitamin B12	a) 170–670 µg/kg IM; b) 16.7 mg/kg IM; c) 5.4% topical spray to 50% of the body; d) 0.5 ml IM	1	na	52 weeks
Hanuman langur (*Semnopithecus entellus*), Chhangani et al. (2001) [30]	30 (i.e. all clinically diseased langurs)	Moderate	a) Tebrub; b) Mebhydrolin	a) 250 mg PO; b) 25 mg PO	a) 30; b) 30	a) 1 day; b) 1 day	9 weeks
	5/30 (i.e. langurs that failed to recover with oral treatment)	Moderate	a) Ivermectin; b) Deltamethrin; c) Chlorpheniramine maleate; d) D.N.S infusion	a) 1 mg/kg SC; b) 2 ml topical spray; c) 10 mg SC; d) 1 l IV	1	na	

^a^Severity of mange prior to treatment

^b^Alphabetized bullet points indicate different medications delivered concurrently

*Abbreviation*: na, not applicable

Post-treatment monitoring periods ranged from 1–74 weeks and were an average of 22.8 weeks and a median of 11 weeks. This is slightly higher than the average and median duration of post-treatment monitoring periods in the studies involving captive wildlife (20.8 and 8 weeks, respectively). Similar to the studies on captive wildlife, two studies were initially unsuccessful at eliminating *S. scabiei* infections from all animals, and some animals required additional treatments [29, 30].

The nature of the post-treatment monitoring periods was generally limited, as multiple studies were either unable or did not attempt to observe or recapture all treated animals [17, 24, 29, 31]. Instead, they based their outcomes upon a few recaptured or remotely observed individuals. This means that their long-term outcomes may not have been truly representative of all animals treated. For instance, Skerratt et al. [29] postulated that the low recapture rate in their study on free-roaming bare-nosed wombats could have been due to mortalities from sarcoptic mange. Furthermore, three studies relied on visual observations from a distance to confirm an absence of infection, rather than direct skin scrapings [9, 17, 30] and in two studies, the post-treatment monitoring period was recorded for less than a month [29, 31].

#### Unsuccessful studies with documented treatment outcomes

The acaricides used in the unsuccessful treatment studies were subcutaneous ivermectin and topical selamectin (Table 4). In these studies, a single injection of ivermectin at a dose between 200 µg/kg and 300 µg/kg was unsuccessful at eliminating infections in moderately to severely-diseased koalas and red foxes [32, 33]. In the other study, a single application of 6.0 mg/kg topical selamectin was able to eliminate mange from three mildly infected San Joaquin kit foxes (*Vulpes macrotis*), although did not recover six foxes with severe disease, who died shortly after treatment. The successfully treated kit foxes were released within 32 days of treatment, although post-release survival outcomes are not reported in the study [19].

**Table 4:** Summary of unsuccessful studies involving the treatment of sarcoptic mange in wildlife

Treated species and reference	No. of animals treated & treatment environment	Severity^a^	Treatment^b^				Outcome
			Drug(s) administered	Dose & delivery method	No. of doses	Treatment interval between doses	
Koala (*Phascolarctos cinereus*), Speight et al. (2017) [33]	1 captive	Severe	a) Ivermectin; b) Enrofloxacin	a) 200 µg/kg SC; b) 10 mg/kg SC	a) 1; b) 3	a) na; b) 1 day	Death within three days of treatment
San Joaquin kit fox (*Vulpes macrotis*), Cypher et al. (2017) [19]	9 captive	Mild or severe	Selamectin	6.0 mg/kg topical application	1	na	Death of 6 foxes within 3 days of capture but recovery of 3 foxes within 29 to 32 days
Red fox (*Vulpes vulpes*), Newman et al. (2002) [32]	15 wild	Unreported	Ivermectin	300 µg/kg SC	1	na	Initial improvement, then gradual death due to overwhelming *S. scabiei* infection

^a^Severity of mange prior to treatment

^b^Alphabetized bullet points indicate different medications delivered concurrently

*Abbreviation*: na, not applicable

#### Studies excluded from further analysis

Ten studies did not provide literature usable for analysis. The most common reason for exclusion was a failure to include a post-treatment monitoring period (see Table 5).

**Table 5:** Studies excluded from analysis and their reasons for exclusion

Treated species and reference	Reason for exclusion
Vicuna (*Vicugna vicugna*), Gomez-Puerta et al. [53]	No treatment outcomes are described
Giraffe (*Giraffa reticulata*), Alasaad et al. [54]	Although reportedly successful on a population level, the study failed to recapture treated individuals and monitor their response to treatment
Capybara (*Hydrochoerus hydrochaeris*), Bernal et al. [55]	No treatment outcomes are described
Agile wallaby (*Macropus agilis*), McLelland et al. [37]	Although reportedly successful on an individual level, no post-treatment monitoring period is described
Gorilla (*Gorilla beringei beringei*), Graczyk et al. [22]	Although reportedly successful on an individual level, the study fails to specify the duration of their post-treatment monitoring period
Iberian ibex (*Capra pyrenaica*), Pérez et al. [56]	Limited treatment outcomes are described and there is no post-treatment monitoring period
Gray wolf (*Canis lupus*), Schultz et al. [17]	Although reportedly successful on an individual level, the diagnosis of sarcoptic mange is not confirmed by skin scrapings
Wild ruminants, Yeruham et al. [16]	Although reportedly successful on a population level, the study fails to specify the duration of their post-treatment monitoring period
Koala (*Phascolarctos cinereus*), Brown et al. [40]	The study does not specify the number of animals treated or recovered
Koala, Barker [38]	Although reportedly successful on an individual level, no post-treatment monitoring period is described

## Discussion

The primary objective of this study was to synthesise and analyse the diverse literature published on the treatment of sarcoptic mange in wildlife from around the world. This involved identifying the qualities of successful treatment strategies in captive and free-roaming wildlife and evaluating their long-term outcomes. This systematic review has found that several successful treatment protocols have been used for captive and free-living wildlife based around the use of subcutaneous ivermectin. In general, ivermectin was used successfully when delivered via subcutaneous injection at a dose between 200–400 µg/kg, between one to four times and at an interval of 10–14 days. Severely-diseased animals also appear to have a better prognosis when given concurrent supportive therapy (such as intravenous fluids, antimicrobials and high-calorie nutrition) [27, 34, 35]. Whilst a single injection of ivermectin at 200–400 µg/kg was reportedly effective at eliminating mange infections in some studies [9, 23, 24], it is recommended that animals receive two to three treatments, 14 days apart, in order to kill *S. scabiei* larvae that emerge from the relatively acaricide-resistant ova [36].

The review also found that the severity of infection often influences the number of treatments required to eliminate infection and the overall success of treatment. For instance, in some studies, severely-diseased or debilitated animals required more doses of ivermectin to eliminate infection than mild or moderately infected animals [23, 26]. Severely-diseased animals were also less likely to recover than mild or moderately parasitised individuals, despite treatment [19, 24, 27, 29, 37]. For these reasons, it is recommended that heavily parasitised animals should be excluded from captive treatment programs aimed at re-introducing animals back into the wild, as demonstrated in the study by Leon-Vizcaino et al. [23]. In situations where the treatment of free-roaming wildlife is being attempted, Skerratt et al. [29] hypothesise that reducing the average intensity of infection to a low level will halt the transmission of *S. scabiei*. To achieve this, Skerratt et al. suggests euthanising moderately to severely parasitised individuals, removing their carcasses from the environment and treating any remaining animals. This technique was used with reported success in the study by Yeruham et al. [16] in which four out of five free-range zoos reported an elimination of sarcoptic mange following euthanasia and removal of severely-diseased individuals from the environment and treatment of remaining animals.

Another pertinent finding was that elimination of infection from clinically-affected individuals within captive populations was only successful in some studies when all in-contact animals were treated simultaneously [25, 38]. Presumably, individuals that are infected sub-clinically act as a source of re-infection for animals more susceptible to clinical manifestations of the disease. Hence, when designing treatment protocols, it is important to treat all in-contact animals, including domestic animals, and ensure humans follow sound biosecurity protocols to avoid becoming a source of infection for captive animals [25, 39].

These findings are expected to help guide veterinarians and wildlife carers in their decision of how best to treat individuals and groups of wildlife brought into captivity for rehabilitation, such as in wildlife hospitals and shelters.

### Post-treatment monitoring periods and long-term outcomes

Based on the findings of this review, there is a low chance that captive wildlife from which mange has been successfully eliminated by treatment with ivermectin or fluralaner will redevelop mange whilst remaining in captivity; only one of the ten studies involving the treatment of wildlife in captivity reported a relapse of mange during their post-treatment observational period [28]. However, information on post-release survival and reinfection rates of wildlife released from captivity remains elusive, as only one of the ten studies attempted monitoring past the point of release from captivity [21]. This prevents the authors from commenting on the likelihood of captive wildlife becoming re-infected with *S. scabiei* following release into the wild. Information from the non-captive wildlife studies suggest it is possible to control infection and reduce the incidence of mange in free-roaming individuals and populations. However, it is uncertain whether infections were truly eliminated from the animals in these studies, or if limited post-treatment monitoring techniques resulted in cases of disease being missed. Consequently, there may have been a higher rate of re-infection and mortalities in free-roaming animals than reported.

### Rationalising treatments and designing a treatment-inclusion criteria

With limited data on post-release survival and re-infection rates, the rationale for bringing wildlife into captivity for treatment may be reasonably questioned. If re-infection rates are high and survival rates are poor post-release, euthanasia and removal of infected bodies from the environment, as demonstrated in a study by Alasaad et al. [40], may be a more appropriate action to relieve suffering and reduce the transmission of disease. One must also question the ethics of treating endemic diseases in free-ranging wildlife with a healthy conservation status; whilst the presentation of sarcoptic mange may raise welfare concerns, it is possible that resultant deaths play a role in natural selection for resistant animals. Granted, where an animal’s conservation status is in question, attempts to treat free-ranging wildlife may be acceptable. One potential method for determining whether treatment is warranted would be to establish a treatment-inclusion criteria. Ideally, the criteria would take into consideration the severity of infection, the likely success of treatment, and post-release survival and re-infection rates. Other important factors for the criteria would include the conservation status of the animal and the likelihood of the animal transmitting infection to another species if left untreated. For example, in the study by Gakuya et al. [17], which focussed on the treatment of a population of threatened cheetahs, wild Thomson’s gazelles also received treatment for sarcoptic mange, despite being locally abundant. This is because they were a reservoir for mites and the primary source of infection for the critically endangered cheetah population. Lastly, the criteria should take into consideration whether the animal is being translocated into a new area in which sarcoptic mange is not endemic, such as a sanctuary or a game park. If this is the case, animals coming from a region where mange is endemic should be treated regardless of whether they show clinical signs of disease, because sub-clinical carriers of the mite have been implicated as the source of outbreaks in multiple captive animal collections [16, 25, 38]. A treatment-inclusion criteria would thereby help to prioritise which species and regions should be targeted for treatment, thus optimising the use of limited resources.

### Limitations of current treatment protocols and suggestions for their improvement

Despite its success, there are disadvantages to the use of multiple injections of ivermectin as the primary method of treatment for sarcoptic mange in wildlife. For instance, it is limited to situations where darting or capture of wildlife is possible. These methods are expensive, as they either require the frequent tracking and immobilisation of wildlife, or maintaining wildlife within facilities for extended periods of time [26]. Captivity is also stressful for wildlife, which may result in stress-related illnesses, treatment failure or death. Furthermore, if release from captivity is not performed properly, animals may die as a result of maladaptation, predation or disease [10]. Whilst the use of pour-on ivermectin may mitigate some of these challenges, its effectiveness in the treatment of sarcoptic mange in wildlife has not been formally documented. In contrast, the macrocyclic lactone, moxidectin, has been under investigation for the treatment of sarcoptic mange in wombats in Australia for several years. For instance, in 2011, Death et al. investigated the pharmacokinetics of injectable moxidectin in healthy southern hairy-nosed wombats [41]. Because moxidectin has a longer half-life than ivermectin in cattle [42] it was thought that wildlife would require less frequent applications of topical moxidectin for elimination of infection. However, this has not been scientifically validated.

In Australia, some research institutions have investigated the efficacy of topical moxidectin for the treatment of mange-infected wombats through remote treatment stations (also known as ‘burrow-flaps’). Their findings are in review and in preparation at the time of this review. The remote treatment method involves installing a large, square plastic flap (usually made of a recycled container lid) on a wire frame directly outside burrow entrances. A small, rectangular hole is cut out of the centre of the flap, and a bottle lid is fixed within the hole, to act as a vessel to hold a small volume of moxidectin. When the wombat leaves the burrow, it tips the flap, which pours the moxidectin out of the bottle lid and onto its back. Treatment protocols vary, but typically involve 500 µg/kg–1,200 µg/kg moxidectin, delivered topically once a week for eight to twelve weeks (Mange Management Inc, personal communication). In 2016, the University of Tasmania undertook a mass-treatment trial of the wombats in Narawntapu National Park, Tasmania, using this treatment protocol. The preliminary findings of the study suggested that the burrow-flap is effective for treating individuals but not an effective method of long-term population-level control of sarcoptic mange in wombats (Scott Carver, personal communication). Furthermore, in a review of the treatment of sarcoptic mange in wombats, Old et al. [43] describe the treatment of a population of wild bare-nosed wombats using remote treatment stations; wombats in the Wolgan Valley, NSW, were treated with 500 µg/kg topical moxidectin once a week for three months during 2011 to 2012. Follow-up spotlighting surveys revealed no change in the mange level of treated wombats and so the treatment method was not substantiated based on the results obtained in the study. The burrow-flap is nonetheless a key example of a relatively inexpensive and non-invasive method of treatment delivery.

Munang’Andu et al. [26] proposed using more potent drugs less frequently as a method of minimally-invasive treatment that promotes ex-situ conservation. It is thought that higher doses of a drug may eliminate infection more rapidly and hence reduce the need for repeated treatments [23, 26]. However, according to Skerratt et al. [28], there is the potential for drug toxicity, especially in animals severely debilitated by mange, because of an alteration in the pharmacokinetic properties of the drug. From this literature review, the risk of toxicity from injectable ivermectin within the range of 200–1000 µg/kg appears to be low: only four studies reported adverse side effects following the delivery of ivermectin, including diarrhoea [9, 18] or death in a compromised individual [19, 20]. However, the link between drug delivery and side effects was tenuous and death may have occurred as a result of the primary disease process or the stress of handling. It is possible that wildlife in the remaining studies experienced drug toxicities as a result of treatment, but these were either unobserved or not documented. Toxicity data could also be extrapolated from studies on domestic animal species to provide more evidence about the risk of dose-related side effects. Nonetheless, the risk of toxicity in any given wildlife species cannot be fully-assessed without conducting formal toxicity trials, especially for higher doses. It must also be stressed that the drugs within the macrocyclic lactone family (such as ivermectin, moxidectin and selamectin) have differing pharmacokinetic properties and toxicity data [42]. Therefore, doses of injectable ivermectin that are considered safe may cause toxicity if the same dose is delivered using another macrocyclic lactone, such as moxidectin. The authors of this review do not recommend extrapolating regimens involving ivermectin to other macrocyclic lactones and *vice versa*.

Yeruham et al. [16] suggested the delivery of ivermectin through feed, as a safer and less stressful alternative to darting or physical restraint and injection. This method proved successful in eliminating sarcoptic mange from four out of five collections of captive ruminants, and a captive American black bear [21], and in eliminating the clinical signs of mange in juvenile wild boar four weeks after treatment [31]. This approach may be useful in situations where animals will eat food laid out by humans, such as permanently captive wildlife, or free-roaming wildlife in a habitat where their natural food source is scarce, such as during a drought [23]. However, Rajkovic-Janie et al. [31] warned that only drugs known to have a broad therapeutic margin should be used, as highly precise drug dosages cannot be ensured when delivering medication through feed to free-ranging animals.

Furthermore, supplementing diseased individuals with high-calorie food, vitamins and minerals, in addition to the provision of an acaricide and other supportive therapy, will likely improve treatment outcomes [34, 44]. This was identified in a recent epidemiological study of sarcoptic mange in wombats, where Martin et al. found that wombats debilitated by mange have heightened energetic demands through heat loss and a raised metabolism.

Finally, new generation parasiticides in the isoxazoline chemical class may also offer promise as treatment alternatives for wildlife. Among these, fluralaner is the most well studied; principally in dogs and cats [45–48]. However, the study by Van Wick et al. has reported successful treatment of *S. scabiei* in an American black bear using a single oral dose of 44 mg/kg [21]. The potential advantage of this group of drugs is the apparent duration of protection conferred against ectoparasites (approximately three months) [49, 50]. Further research is needed to assess the safety, pharmacokinetics and efficacy of isoxazolines in wildlife.

### Limitations of this review

One outcome of this review is the notable lack of randomised controlled studies. Consequently, much of the data must be treated with caution. There is an imperative need for larger and more definitive randomised controlled trials. The reader should also be aware that the conclusions drawn concerning the effectiveness of different treatment protocols may not be applicable for all wildlife species. This is because the metabolism of the macrocyclic lactones varies between species [42], influencing its tissue distribution and half-life. Therefore, different animals may require different drug doses and frequencies of drug delivery in order to achieve elimination of infection. Treatment protocols should be tailored to the wildlife species of interest based on pharmacokinetic data in that species, where available.

## Conclusions

To our knowledge, this review is the first to demonstrate that multiple therapeutic protocols exist for the treatment of sarcoptic mange in wildlife. Ivermectin, injected subcutaneously and delivered multiple times at a dose rate of between 200–400 µg/kg, was found to be the most commonly used acaricide and effective in the treatment of sarcoptic mange in both captive and free-living wildlife. Of the ten studies on captive wildlife that underwent further analysis, only one study documented a recurrence of the clinical signs of mange during their post-treatment monitoring period. However, information on post-release survival and re-infection rates of wildlife released from captivity remains elusive. Of the six studies on free-roaming wildlife that underwent further analysis, all studies reported an absence of the clinical signs of mange during their post-treatment monitoring period. However, poor monitoring techniques and low recapture rates post-treatment means there may have been more treatment failures or relapses of infection than documented. There is an imperative need for larger and more definitive randomised controlled studies. Other potential areas for future research include less stressful alternatives to darting and capture for the delivery of medications to free-roaming wildlife, and the use of the isoxazoline chemical class as a one-off treatment.

## Additional files

**Additional file 1: Table S1.** PRISMA checklist.

**Additional file 2: Text S1.** Full search strategy for Web of Science.

**Additional file 3: Table S2.** Complete data extraction table of all selected studies on the treatment of sarcoptic mange in wildlife from the database search.

## Abbreviations

SC: Subcutaneous injection
IV: Intravenous injection
IM: Intramuscular injection
PO: *per os* (orally)
BID: *bis in die* (twice a day)
