# Supplementary material for: The treatment of sarcoptic mange in wildlife: a systematic review
Source: Parasit Vectors. 2019 Mar 13;12:99. doi: 10.1186/s13071-019-3340-z (PMC6416846; doi:10.1186/s13071-019-3340-z)
Supplement: Supplementary file 2 — Additional file 2: Text S1. Full search strategy for Web of Science. [file 13071_2019_3340_MOESM2_ESM.docx]

**Additional file 2: Full search strategy for Web of Science**

Terms^a^:

| 1. Sarcoptic mange OR sarcop* OR mange OR scabie* |
| --- |
| 1. Wild* OR population* OR native OR indigenous OR local OR animal* OR free-roaming OR free-ranging OR undomesticated |
| 1. Treat* OR therapy OR cure* OR medicate* OR rehabilitate* OR remedy   Strategy: (1 AND 2 AND 3) |

^a^The asterisk (*) is a truncation symbol used to find any character, group of characters, or no character. For instance, *treat** would search for the terms *treats* and *treatment* and *treat.*
