# Supplementary material for: The treatment of sarcoptic mange in wildlife: a systematic review
Source: Parasit Vectors. 2019 Mar 13;12:99. doi: 10.1186/s13071-019-3340-z (PMC6416846; doi:10.1186/s13071-019-3340-z)
Supplement: Supplementary file 3 — Additional file 3: Table S2. Complete data extraction table of all selected studies on the treatment of sarcoptic mange in wildlife from the database search. [file 13071_2019_3340_MOESM3_ESM.docx]

**Additional file 3 Table S2: complete data extraction table of all selected studies on the treatment of sarcoptic mange in wildlife from the database search**

| **Reference & country of publishing** | **Study design** | **Animal(s) treated**^a^ | **Location**^b^ | **Total no. treated**^c^ | **Total no. treated with a specific treatment**^d^ | **Severity**^e^ | **Treatment**^f^ | | | | **Reported recovery rate**^g^ | | | |
| --- | --- | --- | --- | --- | --- | --- | --- | --- | --- | --- | --- | --- | --- | --- |
|  |  |  |  |  |  |  | **Therapeutic agents** | **Dose & delivery method** | **No. of doses** | **Treatment interval between doses** | **0-25%** | **26-50%** | **51-75%** | **76-100%** |
| Van Wick et al. (2018) USA [21] | Case report | American black bear (*Ursus americanus*)  Ursidae | Captive | 1 | 1 | Severe | 1. Fluralaner; 2. Lactated Ringer’s Solution; 3. Hydrogenated iron | 1. 44mg/kg PO (via food); 2. 40mL/kg SC; 3. 10mg/kg IM | 1. 1; 2. 1; 3. 1 | n/a |  |  |  | Y |
| Speight et al. (2017) Australia [33] | Case series | Koala (Phascolarctos cinereus), Phascolarctidae | Captive | 1 | 1 | Severe | 1. Ivermectin; 2. Enrofloxacin | 1. 200µg/kg SC; 2. 10mg/kg SC | 1. 1; 2. 3 | 1. n/a; 2. 1 day | Y |  |  |  |
| Cypher et al. (2017) USA [19] | Case series | San Joaquin kit fox (*Vulpes macrotis*),  Canidae | Captive | 9 | 9 | Mild to severe | Selamectin | 6.0mg/kg topical application | 1 | n/a |  | Y |  |  |
| Kim et al. (2015) Korea [25] | Case series | Mara  (*Dolichotis caviae*),  Caviidae | Captive | 16 | 2 | Moderate | 1. Amitraz; 2. Prednisolone | 1. 0.025% wash; 2. 2mL IM | 4 | 7 days | Y |  |  |  |
|  |  |  |  |  | 16 (2 retreated) | Mild to moderate | 1. Ivermectin; 2. Prednisolone | 1. 400µg/kg; 2. 2mL IM | 1. 4; 2. 4 | 1. 7 days; 2. 7 days |  |  |  | Y |
| Kido et al. (2014) Japan [27] | Non-randomised controlled trial | Raccoon dog (*Nyctereutes procyonoides*), Canidae | Captive | 225 | 68 | Moderate to Severe | Ivermectin | 400µg/kg SC | 2 | 14 days |  | Y |  |  |
|  |  |  |  |  | 157 | Moderate to Severe | 1. Ivermectin; 2. Cephalexin; 3. Lactated Ringer’s Solution | 1. 400µg/kg SC injection; 2. 20mg/kg PO or IV BID; 3. IV infusion | 1. 3; 2. 7; 3. 1 | 1. 14 days; 2. 1 day; 3. n/a |  |  | Y |  |
| Ruykys et al. (2013) Australia [24] | Cohort study and case report | Southern hairy-nosed wombat (*Lasiorhinus latrifrons*), Vomatidae | Captive and wild | 5 (2 captive and 3 wild) | 5 | Mild or severe | Ivermectin | 200µg/kg SC | 1 | n/a |  |  | Y |  |
| Gomez-Puerta et al. (2013) Peru [53] | Case report | Vicuna (*Vicugna vicugna*), Camelidae | Wild | 200 | 200 | None to moderate | 1. Ivermectin; 2. ‘Vitamins’ | 1. Unknown dose injected; 2. Unknown dose injected | 1 | n/a | Outcomes unreported | | | |
| Bernal et al. (2011)  Spain [55] | Non-randomised, controlled trial | Capybaras (*Hydrochoerus hydrochaeris*),  Caviidae | Captive | 10 | 10 | Unknown | Ivermectin | 200µg/kg SC | 1 | n/a | Outcomes unreported | | | |
| Gakuya et al. (2012) Kenya [17] | Cross-sectional study | Cheetah (*Acinonyx jubatus*), felidae; Thomson's gazelle (*Eudorcas thompsonii*), bovidae; sheep (*Ovis* aries) Bovidae; Cattle (*Bos* indicus), bovidae; goat (*Capra* hircus), Bovidae; dog (*Canis lupus familiaris*), candiae; Wildebeest (*Connochaetes taurinus*), bovidae; Lion (*Panthera leo*), Felidae | Wild | Large numbers (exact figures unknown) | Unknown | Mild to severe | Ivermectin | 200µg/kg SC | 1 | n/a |  |  | Y |  |
| Alasaad et al. (2011) Kenya [54] | Case series | Giraffe  (Giraffa reticulata), Giraffidae | Wild | 5 | 5 | Mild to moderate | 1. Ivermectin; 2. ‘Broad spectrum antibiotic’ | 1. 300µg/kg SC; 2. Topical spray | 1 | n/a |  |  |  | Y |
| Munang’Andu et al. (2010) Zambia [26] | Case series | African buffalo (*Syncerus caffer*), Bovidae | Captive | 77 | 77 | Mild to severe | Ivermectin | 200µg/kg SC | 4 | 30 days |  |  |  | Y |
| Menzano et al. (2008) Italy [20] | Case report | Roe deer  (*Capreolus capreolus*), Cervidae | Captive | 3 | 3 | Moderate to severe | Ivermectin | 300µg/kg SC | 3 | 15 days |  |  | Y |  |
| McLelland et al. (2005) Australia [37] | Case series | Agile wallaby (*Macropus agilis*),  Macropodidae | Captive | 3 | 1 | Moderate to severe | 1. Ivermectin; 2. Long-acting corticosteroid | 1. Unknown dose injected; 2. Unknown dose injected | Unknown | Unknown | Y |  |  |  |
|  |  |  |  |  | 2 (1 retreated) | Moderate to severe | Selamectin | 45mg topically | 3 | 7 days |  |  |  | Y |
|  |  |  |  |  | 1 | Mild | Ivermectin | 300µg/kg SC injection | 2 | 14 days |  |  |  | Y |
| Rajkovic-Janje et al. (2004)  Croatia [31] | Case series | Wild boar (*Sus scrofa*), Suidae | Wild | 750 | 750 | Unknown | Ivermectin | Approximately 100µg/kg PO delivered via a 0.6% ivermectin food formula | 7 | 1 day |  |  |  | Y |
| Skerratt et al. (2004) Australia [29] | Non-randomised controlled trial | Common wombat (*Vombatus ursinus*), Vombatidae | Wild | 5 | 5 | Mild to moderate | 1. Ivermectin; 2. Amitraz | 1. 400-800µg/kg SC; 2. 0.025% topical wash | 1. 2; 2. 1 | 1. 28 days; 2. n/a |  |  |  | Y |
|  |  |  |  |  | 1  (retreated) | Mild | Ivermectin | 800µg/kg SC injection | 2 | 10 days |  |  |  | Y |
| Skerratt et al. (2003) Australia [28] | Non-randomised controlled trial | Common wombat (*Vombatus ursinus*), Vombatidae | Captive | 7 | 2 (Experiment One) | Mild to severe | 1. Ivermectin; 2. Procaine penicillin; 3. Benzaine penicillin | 1. 300µg/kg SC; 2. 15mg/kg IM; 3. 11mg/kg IM | 1. 3; 2. 1; 3. 1 | 1. 10 days; 2. n/a; 3. n/a |  |  |  | Y |
|  |  |  |  |  | 7  (Experiment Two) | Mild to severe | 1. Ivermectin; 2. Procaine; penicillin 3. Benzaine penicillin | 1. 300µg/kg SC; 2. 15mg/kg IM; 3. 11mg/kg IM | 1. 3; 2. 1; 3. 1 | 1. 10 days; 2. n/a; 3. n/a |  |  | Y |  |
|  |  |  |  |  | 3  (Retreated from Experiment Two) | Mild | Ivermectin | 300µg/kg SC | 3 | 10 days |  |  |  | Y |
| Newman et al. (2002) England [32] | Retrospective cohort study | Red fox (*Vulpes Vulpes*),  Canidae | Wild | 15 | 15 | Unreported | Ivermectin | 300µg/kg SC injection | 1 | n/a | Y |  |  |  |
| Kalema-Zikusoka et al. (2002) Uganda [9] | Case series | Mountain gorillas (*Gorilla beringei beringei*), Hominidae | Wild | 3 | 3 | Moderate to severe | 1. Ivermectin; 2. Long-acting streptopenicillin; 3. Oxytetracycline gentian violet spray; 4. Ferrum 10%; 5. Vitamin B12 | 1. 170-670µg/kg IM; 2. 16.7mg/kg IM; 3. 16mg/kg IM; 4. 5.4% topical spray; 5. 0.5mL IM | 1 | n/a |  |  |  | Y |
| Graczyk et al. (2001) Uganda [22] | Case series | Mountain gorillas (*Gorilla beringei beringei*), Hominidae | Wild | 5 | 5 | Moderate to severe | 1. Ivermectin; 2. Penicillin; 3. 0.9% NaCl solution | 1. 200µg/kg SC; 2. 4.0mL SC; 3. 1L SC | Unknown | Unknown |  |  |  | Y |
| Leon-Vizcaino et al. (2001) Spain [23] | Non-randomised controlled trial | Spanish ibex (*Capra pyrenaica*), Bovidae | Captive | 15  (For experimental treatment) | 3 | Moderate | Ivermectin | 400µg/kg SC | 1 | n/a |  |  |  | Y |
|  |  |  |  |  | 3 | Moderate | Ivermectin | 400µg/kg SC or superficial IM by rifle dart | 1 | n/a |  |  |  | Y |
|  |  |  |  |  | 3 | Moderate | Ivermectin | 200µg/kg SC | 1 | n/a |  |  |  | Y |
|  |  |  |  |  | 3 | Severe | Ivermectin | 200µg/kg SC | 2 | 14 days |  | Y |  |  |
|  |  |  |  |  | 3 | Severe | Ivermectin | 400µg/kg SC | 2 | 14 days |  |  | Y |  |
|  |  |  |  | 63  (For the sanitation program) | 17 | None to mild | 1. Phoxim; 2. Ivermectin; 3. Vitamins and minerals | 1. 500mg/L; topical spray 2. 400µg/kg SC; 3. PO (via food) | 1. 1; 2. 1; 3. Ongoing | 1. n/a; 2. n/a; 3. 1 day |  |  |  | Y |
|  |  |  |  |  | 32 | Mild to moderate | 1. Phoxim; 2. Ivermectin; 3. Vitamins and minerals | 1. 500mg/L topical spray; 2. 200-400µg/kg SC or superficial IM by rifle dart; 3. PO (via food) | 1. 1; 2. 1-4; 3. Ongoing | 1. n/a; 2. 14 days; 3. 1 day |  |  |  | Y |
|  |  |  |  |  | 14 | Severe | Euthanised | n/a | | | | | | |
| Chhangani et al. (2001) India [30] | Case series | Hanuman Langur *(Semnopithecus entellus*),  Cercopithecidae | Wild | 30 | 30 | Moderate | 1. ‘Tebrub’; 2. Mebhydrolin | 1. 250mg PO (via food); 2. 0.25mg PO (via food) | 1. 30; 2. 30 | 1. 1 day; 2. 1 day |  |  | Y |  |
|  |  |  |  |  | 5 (Retreated) |  | 1. Ivermectin; 2. Deltamethrin; 3. Chlorpheniramine maleate; 4. D.N.S infusion | 1. 20mg SC; 2. 2mL topical spray; 3. 10mg SC injection; 4. 500mL IV | 1. 1; 2. 1; 3. 1; 4. 1 | n/a |  |  |  | Y |
| Lavin et al. (2000) Spain [51] | Non-randomised controlled trial | Chamois (*Rupicapra rupicapra*), Bovidae | Captive | 2 | 2 | Mild to moderate | Ivermectin | 200µg/kg SC injection | 2 | 15 days |  |  |  | Y |
| Pérez et al. (1999) Spain [56] | Cross-sectional study | Spanish ibexes (*Capra pyrenaica*),  Bovidae | Captive | 20 | 20 | Mild to severe | 1. Ivermectin; 2. Lindane | 1. 200µg/kg SC; 2. 1% topical application | 1. 1; 2. 1 | n/a | Outcomes unreported | | | |
| Schultz et al. (1999) USA [18] | Case report | Gray wolf (*Canis lupus*), Canidae | Captive | 1 | 1 | Moderate | 1. Ivermectin; 2. Etoxazole | 1. 2,000µg/kg SC; 2. Topical sponge-on solution | 1. >1; 2. 1 | 1. Unknown; 2. n/a |  |  |  | Y |
| Little et al. (1998) USA [52] | Non-randomised controlled trial | Red fox (*Vulpes Vulpes*),  Canidae | Captive | 5 | 5 | Severe | Ivermectin | 400µg/kg SC | 2 | 14 days |  |  |  | Y |
| Yeruham et al. (1996) Israel [16] | Case series | Mountain gazelle (*Gazella gazelle*), Thompson’s Gazelle (*Gazella thompsoni*), Nubian ibex (*Capra nubiana*), Barbary sheep (*Ammotragus lervia*), Gnu (*Connochaetes taurinus*), Eland (*Taurotragus oryx*), Arabian oryx (*Oryx leucoryx*); all Bovidae. | Open-range zoological gardens | Five zoological gardens with an unknown number of animals | All animals across the five zoos | Mild to moderate | Ivermectin | 200µg/kg PO (via food) for three days in a row at a time | 3 | 14 days |  |  | Y |  |
|  |  |  |  |  |  | Severe | Euthanasia | n/a | | | | | | |
| Brown et al. (1982) Australia [38] | Case series | Koala (Phascolarctos cinereus),  Phascolarctidae | Captive | A colony with an unknown number of koalas | All koalas within the colony | Mild to severe | Amitraz | 0.025% wash | 2 | 10 days |  |  | Y |  |
| Barker (1974) Australia [39] | Case report | Koala (Phascolarctos cinereus), Phascolarctidae | Captive | 1 | 1 | Severe | Malathion | 0.2% emulsion topically | 3 | 10 days |  |  |  | Y |

^a^Common name, *(Scientific* name), taxonomic family

^b^Treatment environment for the duration of the study (ie. in the wild or in captivity)

^c^The total number of animals that received treatment for sarcoptic mange within the study. This excludes any untreated animals within the control group of experimental studies

^d^The total number of animals that received the subsequent specified treatment regimen, different to other animals in the study

^e^Severity of mange prior to treatment

^f^Alphabetized bullet points indicate different medications delivered concurrently

^g^Where ‘Y’ indicates the percentage of animals that reportedly recovered with treatment

*n/a*: *Not applicable*
